# Variability of iodine concentrations in the human placenta

**DOI:** 10.1038/s41598-019-56775-3

**Published:** 2020-01-13

**Authors:** Kristof Y. Neven, Cédric B. D. Marien, Bram G. Janssen, Harry A. Roels, Nadia Waegeneers, Tim S. Nawrot, Ann Ruttens

**Affiliations:** 1 Centre for Environmental Sciences, Hasselt University, Agoralaan Gebouw D, 3590 Diepenbeek, Belgium; 2 SD Chemical and Physical Health Risks, Sciensano, Leuvensesteenweg 17, 3080 Tervuren, Belgium; 3 Louvain Centre for Toxicology and Applied Pharmacology, Université catholique de Louvain, E. Mounierlaan 53, 1200 Brussels, Belgium; 4 Department of Public Health & Primary Care, Leuven University, Kapucijnenvoer 35, 3000 Leuven, Belgium

**Keywords:** Epidemiology, Endocrinology, Mass spectrometry

## Abstract

Iodine is an essential trace element, necessary for the production of thyroid hormones, which play a key role in optimal foetal growth and (neuro-) development. To date, iodine deficiency remains a health burden in many countries. We investigated the variability of placental iodine concentrations within and between individuals. We used 20 mother-neonate pairs from the ENVIR*ON*AGE birth cohort, took samples at three standardized locations of the placentas, pooled and digested them, and determined the iodine concentrations using an ICP-MS method as an alternative for the Sandell-Kolthoff method. The variability between and within the three sample regions was calculated using the intra-class correlation coefficient (ICC) from the variance components of mixed models. With the Friedman test, the differences between placental biopsies were assessed. The ICC showed a higher between-placenta (68.6%) than within-placenta (31.4%) variability. Subsequently, we used our optimized method to determine iodine concentrations in 498 mother-neonate pairs, which averaged 26.1 *μ*g/kg. For 96 mothers, the urinary iodine concentrations were also determined, which showed no correlation with the placental iodine storage, as was expected. Future studies are necessary to explore the effects of these placental iodine concentrations in relation to health outcomes of mother and child at birth and later in life.

## Introduction

During gestation, the placenta acts as an important gatekeeper between the foetal and the maternal environment. This crucial organ is able to prevent harmful substances from reaching the foetus and allows nutrients, like iodine, to be transported from mother to child. This trace element plays a vital role in the production and regulation of thyroid stimulating hormone (TSH) and the thyroid hormones tri-iodothyronine (T3) and thyroxine (T4), which are essential for optimal foetal growth and (neuro)development^1^. These thyroid hormones are produced by the maternal thyroid gland, bind to plasma proteins for transportation throughout the body, cross the placenta and subsequently reach the foetus^2^. Both hormones play an essential role in the foetal neurogenesis, as well as normal development and differentiation of numerous cell types throughout the body^3^. Both inadequate or excess amounts of iodine can lead to a decrease in the thyroid hormone levels in both mother and foetus, which could impair the neurocognitive development^4^. In the first trimester of pregnancy, the foetus is unable to produce these hormones itself, and relies entirely on the maternal thyroid hormone stores^5,6^. In the later stages of pregnancy, the role of the mother is to maintain an adequate iodine supply to the foetal thyroid gland^7^.

The diet is the most important source of iodine for humans. According to the World Health Organization (WHO), the recommended daily iodine intake for the pregnant and lactating population should range between 150 to 249 *μ*g per day to ensure iodine sufficiency^8^. While numerous iodine supplementation programmes have been implemented over the last decade, an estimated 2.2 billion individuals (i.e. 38% of the world’s population) are affected by iodine deficiency^9^, and it still remains as the leading cause of preventable mental retardation around the world^8^. It has been proposed that the placenta plays a role in the uptake and reserve-storage of iodine^10^, which may represent an adaptive mechanism protecting the foetus from inadequacies in maternal iodine intake.

The primary elimination route for iodine in humans is the renal clearance, and the urinary iodine concentration (UIC) reflects very recent dietary iodine intake^11^. Several studies have used spot urine samples or 24-h UIC to assess the maternal iodine status during pregnancy^12–15^. However, for such purpose, this approach is not without limitations. For example, the UIC does not reflect long-term iodine deficiency, as it varies from day to day and even within a given day, depending on the iodine content of recently consumed food. This often results in misclassification of the maternal iodine status^16^. Therefore, Burns and colleagues^1,10^ recently proposed that iodine concentrations in the placenta itself can be used as a long-term biomarker for iodine consumption during pregnancy.

Various techniques have been described for the determination of iodine concentrations in biological samples and a review of these techniques is provided by Shelor and colleagues^17^. The oldest and likely most widely used technique is the catalytic Sandell-Kolthoff method^18^, particularly in urine samples. As such it has been applied worldwide for the detection of iodine deficiency in populations^17,19^. For placental tissue, some studies used an overnight drying and incineration technique followed by iodine determination with the Sandell-Kolthoff method^1,20^. The National Health and Nutrition Examination Survey was the first study to use ICP-MS for measurements of UIC, and acknowledges several advantages over Sandell-Kolthoff, such as ruggedness, reliability and the speed of the method^17,21^. ICP-MS reportedly has a lower detection limit, a better precision and better accuracy^17,22^. Moreover, in contrast to the Sandell-Kolthoff method, ICP-MS does not suffer from interferences by organics like thiocyanate (i.e. an important component of cigarette smoke), metals like silver and mercury, or nitrite and ascorbic acid^17^. In many areas of research, ICP-MS is increasingly recognised for reliable and rapid detection of low element concentrations, providing high sensitivity and the advantage of multi-elemental detections^21,23,24^.

At the European level, a CEN standardized operating procedure exists (CEN EN 15111:2007)^25^ describing a method for the determination of iodine in various types of food samples, which makes use of tetramethylammonium hydroxide (TMAH) for alkaline iodine extraction, followed by ICP-MS detection. A similar method was reported by Fecher and colleagues^26^. While acidic mineralizations, which are commonly used to digest sample matrices and extract trace elements prior to ICP-MS detection, hold - in case of iodine determination - the risk of a loss of analyte due to the formation of volatile HI or I_2_^27^, while these volatilisations are avoided under alkaline conditions.

In the current study, we present an optimised method for the determination of iodine concentrations in placental tissue based on TMAH extraction followed by ICP-MS detection, including the presentation of method performance characteristics. This is in contrast with previous studies reporting iodine concentrations in placentas, where such performance data were missing. The method was used to (1) check the intra-placental variations of iodine in 20 mother-neonate pairs, and (2) determine the placental iodine concentrations of 498 mother-neonate pairs.

## Results and Discussion

### Determination of the dry weight percentage of the placental samples

The dry weight percentage, determined in ten randomly selected placentas, ranged between 18.0% ± 1.0% and 21.8% ± 1.0%, with an average of 20.1% ± 1.0%. Within a single placenta, the standard deviations between regions ranged from 0.04% to 1.4%. Because of these limited variations, we concluded that the influence of the water content of the samples on the measured iodine concentrations had not to be taken into account in further result interpretation and that the placental iodine concentrations of the present study could best be expressed on a wet weight basis (w/ww).

### Method performance characteristics

#### Limit of detection (LOD) and limit of quantification (LOQ)

The LOD and LOQ in solution of the iodine measurements were respectively calculated as three and ten times the standard deviation of 10 procedure blanks. The LOD was 0.012 *μ*g/L and LOQ 0.040 *μ*g/L in solution, which corresponds to an LOQ of 4 *μ*g/kg in the placenta matrix after taking into account the 100× dilution factor of the samples. Because this LOQ is approximately three times lower than the lowest placental iodine concentration observed in this study, the sensitivity of the method is largely sufficient in the present context.

#### Trueness and recovery

The mean spike recovery at various levels ranged between 102% and 107% of the expected concentrations (see Table 1).

**Table 1:** Three placental samples with known iodine concentrations (i.e. their iodine concentrations were determined prior to spiking) were spiked to three different levels: 75 *μ*g/kg, 150 *μ*g/kg, and 300 *μ*g/kg.

Spike level	Spike concentration		Recovery (%)
	Expected (*μ*g/kg)	Measured (*μ*g/kg)	
1	75	80.4 ± 1.6	107.2
2	150	155.3 ± 1.6	103.5
3	300	305.4 ± 1.6	101.8

Recovery was within 10% of the expected concentration.

Throughout all experimental batches, the average measured I_tot_ concentration of the standard reference material (SRM) 1577 was 294.1 ± 12.5 *μ*g/kg. Compared to the iodine reference value of the SRM 1577 certificate for the MS technique (280 ± 10 *μ*g/kg), the mean measured value in our study was 5% higher. This corroborates with the results of the spiking experiments, which were in agreement with the theoretical values. As such, the accuracy of the method was considered satisfactory.

Based on the observed within-day variations and between-day variations of the results of the SRM 1577 material, the repeatability standard deviation (s_r_) of the method was calculated to be 2.1%, and the between-day standard deviation (s_d_) to be 4.1%. This resulted in a within lab reproducibility (s_ip_) of the method of 4.6%. Calculations for the placental sample analyzed in three independent replicates on three different days resulted in the following values: s_r_ = 1.8%, s_d_ = 2.2%, s_ip_ = 2.9%, which is in line with the results obtained on the reference material.

The method described in the present study (based on TMAH extraction, followed by ICP-MS measurement) has some advantages over the previously described Sandell-Kolthoff reaction^1^, or the acidic extraction of iodine, followed by ICP-MS^28^. First, in comparison with the method proposed by Burns *et al.*^1^, the sample preparation time is shorter and more energy efficient: we digested samples for 3 hours at 90 °C in a TMAH solution, compared to overnight drying and subsequent incineration for 3 hours at 600 °C. Second, ICP-MS is a highly sensitive and precise method for measuring iodine compared to other methods^17^. In comparison with the performance characteristics for various methods and matrices (other than placental tissue) listed by Shelor and colleagues^17^, the LOQ of our method is among the lowest of all values listed and, like the other ICP-MS data cited in that review, our precision values are low compared to many of the cited Sandell-Kolthoff based methods for which relative standard deviations up to 15% were noted. Spike recovery did not show clear differences among methods and our results were in line with those reported previously. Furthermore, previous studies have shown that ICP-MS provides a more precise approach to determine iodine in complex matrices, like breast milk^29^.

Some uncertainty remains regarding the suitability of acid mineralizations (with HNO_3_) as a sample preparation method for placental tissues prior to iodine measurements with ICP-MS as performed by Peng and colleagues^28^. Due to the risk for formation of volatile compounds such as HI or I_2_, iodine losses may occur^26^. Unfortunately, neither that recent study^28^, nor the study by Burns and colleagues^1^ provide information on the performance characteristics of the method they used, which does not permit a sound evaluation of the accuracy of their methods.

### Intra- versus interplacental differences in iodine concentrations

**Figure 1:**
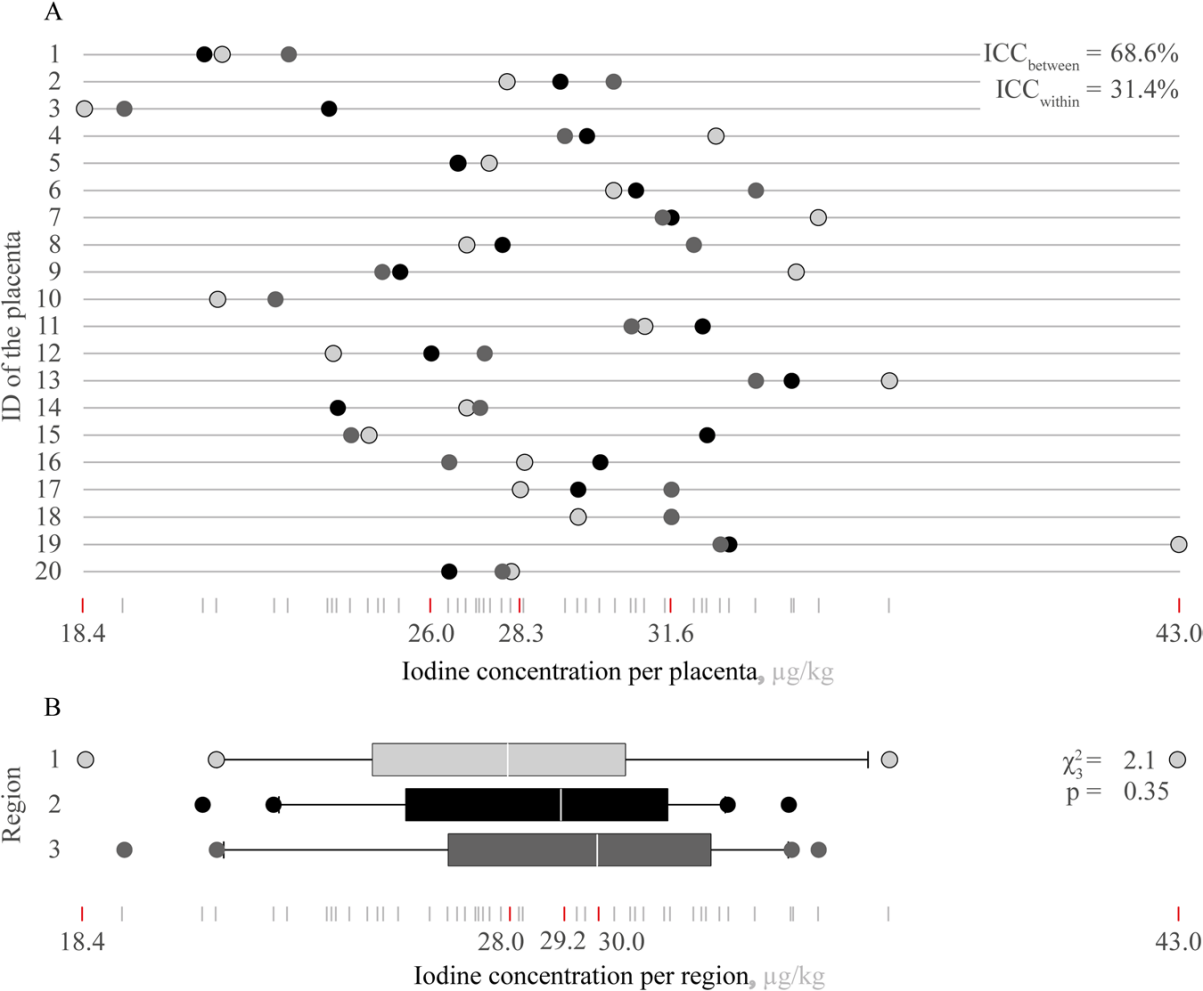
Iodine levels of three biopsy regions of twenty randomly selected placentas. In Panel (A) the placental iodine concentrations of the three regions of each placenta (ID 1 to 20) are plotted. The min-max range was 18.4–43.0 *μ*g/kg, and the 25^th^, 50^th^, and 75^th^ quartiles are 26.0, 28.3, and 31.6 *μ*g/kg, respectively. In Panel (B) the 20 iodine concentration values per biopsy region are shown. The median values for the regions 1, 2, and 3 were 28.0, 29.2, and 30.0 *μ*g/kg, respectively. There was no statistically significant difference between the regions (${\chi }_{3}^{2}$ statistic = 2.1; p = 0.35).

The median placental iodine level in the twenty placentas was 28.3 *μ*g/kg (range: 18.4 *μ*g/kg to 43.0 *μ*g/kg). The intra-class correlation coefficient (ICC) showed that the between-placenta variability (68.6%) was higher than the within-placenta variability (31.4%; p = 0.004; Fig. 1A). Moreover, Friedman statistic showed that no statistically significant difference was observed between iodine concentrations in different regions of the placenta (i.e. ${\chi }_{3}^{2}$ statistic = 2.1; p = 0.35; Fig. 1B).

These statistical results suggest that one single sample of one single region of a placenta reliably reflects the iodine concentration of the whole placenta. However, we assumed the differences between the regions of the individual placentas to be considerable. The maximum difference within one single placenta was 10.3 *μ*g/kg. For this reason as well as to take into account any residual regional differences, we ultimately decided for the determination of the iodine concentration in the 498 placentas of the Environmental Influences *ON* Early Ageing (ENVIR*ON*AGE) birth cohort, to analyze a pooled sample of each placenta, consisting of equal sample amounts of each of the three regions.

### Study population characteristics

The full characteristics of the 498 mother-neonate pairs of the current study are shown in Supplementary Table S1. Mothers were on average 29.5 years old (SD 4.4). The mean maternal pre-pregnancy BMI was 24.6 kg/m^2^ (SD 4.8), and mothers gained 13.8 kg (SD 5.8) during their pregnancy. The majority of them obtained a higher education (54.4%), and 320 participants never smoked (64.4%). During pregnancy, few mothers had hypothyroidism (2.4%), and only 4 were diagnosed with hyperthyroidism (0.8%). Of the neonates, 261 (52.4%) were primiparous, and 51.4% were boys. They had a mean birth weight of 3452 g (SD 436), and had a mean length of 50.3 cm (SD 1.9). 87.7% was of European descent (i.e. at least two European grandparents).

### Iodine concentration in 498 placentas of the ENVIR*ON*AGE birth cohort

**Figure 2:**
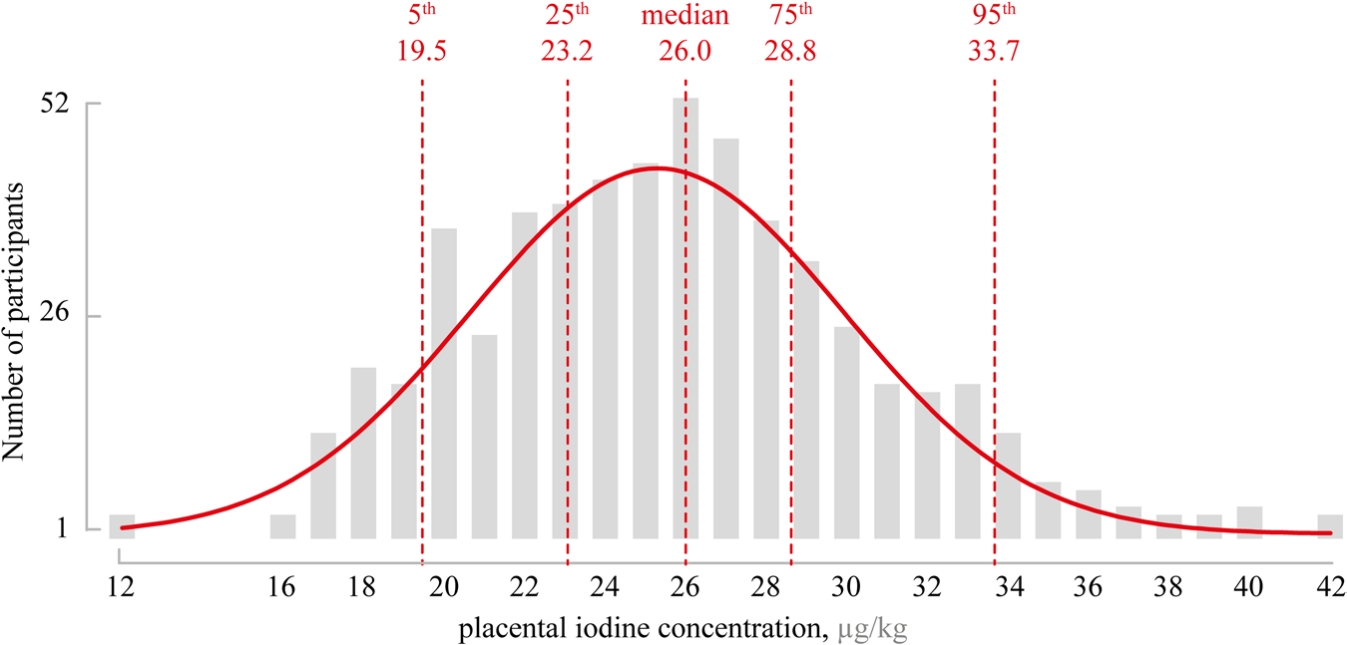
Histogram of the normally distributed placental iodine concentrations in the 498 participants of the ENVIR*ON*AGE birth cohort. Gaussian distribution with a robust fit is shown in red. The lowest and highest observed iodine concentrations were 12.4 and 42 *μ*g/kg, respectively. The median concentration was 26.0 *μ*g/kg.

The mean iodine concentration in the placentas of the ENVIR*ON*AGE birth cohort was 25.6 *μ*g/kg (SD 4.6 *μ*g/kg), ranging from 12.4 *μ*g/kg to 42.0 *μ*g/kg (see Fig. 2). The 10^th^ and 90^th^ percentiles were 21.0 *μ*g/kg and 31.9 *μ*g/kg, respectively. These levels are in line with the observations of Burns and colleagues^1^, who reported an average concentration of 34.0 *μ*g/kg in placental samples collected from an Irish population, using the Sandell-Kolthoff colorimetry. It must be noted that the majority of these pregnant Irish women was iodine deficient, as evaluated by the UIC. In contrast, reported levels of iodine in placentas from mother-neonate pairs of the Liaoning Province in northeastern China (a known iodine sufficient region) were 10 to 20 times higher than those from our study^28^. In that study, iodine concentrations were measured by ICP-MS after acid mineralization (HNO_3_ + H_2_O_2_) in a microwave oven. It is important to note that differences in the applied method, without availability of performance data of all methods, limits and complicates the comparison and interpretation of the observed differences in iodine concentration. In our study, no significant differences in the placental iodine content were observed for maternal age (p = 0.42), maternal education (p = 0.09), gestational thyroid status (p = 0.08), neonates’ sex (p = 0.28), parity (p = 0.34), or ethnicity (p = 0.93). Data are presented in Table 2 and Supplementary Figure S1. These observations are in line with previous studies^30,31^, in which the investigators also found no difference in iodine intake according to parental age, educational level, child’s sex, or parity. Furthermore, we observed no difference in iodine storage between neonates of different ethnicity. This is in contrast to results from Caldwell and colleagues^32^. However, the authors noted that a larger sample size would have been favourable as it would be more representative for race and ethnicity. Furthermore, their racial and ethnic grouping has been made on different attributes compared to our population, which might yield different results.

**Table 2:** Population characteristics of the 498 mother-neonate pairs.

Characteristics	Mean ± SD or number (%)
**Maternal**	
Age, years	29.5 ± 4.4
Maternal education^a^	
*Low*	64 (12.9%)
*Middle*	163 (32.7%)
*High*	271 (54.4%)
Gestational thyroid status	
*Normal*	482 (96.8%)
*Hypothyroidism*	12 (2.4%)
*Hyperthyroidism*	4 (0.8%)
**Neonatal**	
Sex	
*Male*	256 (51.4%)
*Female*	242 (48.6%)
Ethnicity^b^	
*European*	437 (87.7%)
*Non-European*	61 (12.3%)
Parity	
*1*	261 (52.4%)
*2*	172 (34.5%)
≥*3*	65 (13.1%)

^a^Maternal education was coded as “low” (no diploma or primary school), “middle” (high school) and “high” (college or university degree).

^b^Classification of ethnicity is based on the native country of the neonates’ grandparents as either “European” (at least two grandparents were European) or “non-European” (at least three grandparents were of non-European origin).

Because most available data on the iodine status of populations concerns UIC, we also measured iodine concentrations in a subset of 96 spot urine samples, collected the day after delivery. The results of these measurements are reported in Supplementary Fig. S2. As expected, no correlation was observed between spot UIC results and the placental iodine load. While the former reflects short-term exposure (highly influenced by recent diet and therefore highly variable), the latter is more representative for long-term exposure. According to the WHO guidelines for pregnant women, a median urinary iodine concentration of 150–249 *μ*g/L indicates an adequate iodine nutrition during pregnancy, while values less than 150 *μ*g/L are considered insufficient^33^. In a recent study by Vandevijvere and colleagues^12^, it was shown from the UIC measurements that most pregnant women in Belgium are iodine deficient. They reported a median UIC of 131.0 *μ*g/L, which is higher than the median (in 96 samples) of the ENVIR*ON*AGE cohort of 55.7 *μ*g/L (or 69 µg/g creatinine). Although UIC tends to be lower shortly after delivery than during pregnancy^34,35^, these results suggest that over a period of four to six years, iodine deficiency further increased in the Belgian pregnant population. A similar trend has been observed in the United States National Health and Nutrition Examination Survey (NHANES) where each cycle the UIC decreased: from 181 *μ*g/L in 2000^21^ to 125 *μ*g/L in 2005–2008^32^. More data are needed to confirm this Belgian trend. In any case, both the placental iodine concentrations and UIC reported in the present study, are indicative for concentrations found under iodine deficiency.

### Strengths and limitations

Our study has some strengths and weaknesses. First, the samples were drawn from three standardized regions of the placenta, instead of homogenizing the entire placenta, which would yield a placental homogenate containing placental blood, fragments of blood vessels, and membranes. Nonetheless, reproducibility and variation between the three regions were adequately checked, and the variation between single regions within a single placenta was low. Moreover, a pooled sample of the various sampled regions was analyzed to obtain a more representative result. Second, as the current WHO threshold values for iodine are only based on the UIC values and not on placental iodine concentrations, our data cannot be used to determine the true extent of iodine deficiency within the current birth cohort. However, their value is in provision of an accurate and stable representation of long-term iodine intake throughout the entire pregnancy, which in a later step could be related to health outcomes of both mother and child. Placental threshold values as such are of little importance with regard to screening and prevention of iodine deficiency during reproductive phase of life, as iodine deficiency should already be addressed before and during pregnancy. However, determining the placental iodine concentrations can be important for biomonitoring and preventive medicine as a long-term biomarker of gestational iodine load. It is a valuable tool to diminish exposure misclassifications, which are more likely to occur when measuring urinary iodine concentration. A final, major strength of the current study is that our findings can be extrapolated to the general Belgian population, as our study population is representative for the segment of the Belgian population in the pregnancy period or reproductive phase of life (Supplementary Table S2)^36^.

## Conclusions

In the present study, we present an optimized method for the determination of iodine concentrations in placental tissue samples based on extraction with TMAH followed by ICP-MS measurement. Our method of tissue sampling and subsequent measuring of iodine concentration provides a novel approach, with a high precision and accuracy, to determine the concentration of iodine accumulated during gestation in the placenta. In contrast to other existing studies reporting iodine concentrations in placentas, we detail for the first time the performance characteristics of such a method. In addition to the validated analytical protocol, we applied a standardised sampling protocol and we were able to show that there was no significant difference in iodine levels between biopsies taken at three standardised regions of the placenta. Nevertheless, we used a pooled sample of the different regions to further reduce variation of the results. The proposed method was successfully applied for the assessment of placental iodine levels in 498 mother-neonate pairs of the ENVIR*ON*AGE birth cohort. The mean placental iodine concentration in the current study was 25.6 *μ*g/kg (SD 4.6), with a range between 12.4 *μ*g/kg and 42.0 *μ*g/kg. While these data are in line with previous reports which used different sampling and analytical strategies, this is the first study to provide performance characteristics on the given method. Furthermore, we have sampled and measured placental iodine levels for a large number of participants. No correlation was observed between the placental and urinary iodine concentrations.

We believe that placental iodine concentration could be used as a proxy of long-term iodine consumption of the mother and presents some advantages over UIC, not the least because the UIC, even when 24 h collections are used, is more susceptible to iodine concentrations in foods consumed the day(s) before urine sampling. In this regard, placental concentrations of iodine would reflect a more stable overall iodine status because of the long-term accumulation and the uptake mechanism into placental tissue via the sodium iodide symporter^37,38^. Future studies, however, are necessary to explore the effects of placental iodine concentrations in relation to health outcomes of the children at birth and later in life.

## Materials and Methods

### Study design

In 2011, the ENVIR*ON*AGE birth cohort was initiated as a collaboration between Hasselt University (Hasselt, Belgium) and the East-Limburg Hospital (Genk, Belgium), and recruits pairs of mothers and neonates (singleton births only) at birth. The study protocol was approved by the Ethics Committee of Hasselt University and the East-Limburg Hospital and recruitment occurred in accordance to the Helsinki declaration. Written informed consent was obtained from all participating mothers.

The inclusion criteria were mothers who provided informed consent and able to fill out questionnaires in Dutch, while planned caesareans were excluded. Midwives recorded the main reasons for non-participation (such as, complications during labour, communication barriers, or failure to ask participation)^39^. We obtained medical and lifestyle data like maternal age, education, smoking status, ethnicity, and the neonate’s parity via questionnaires^39^. A comprehensive description of the cohort and full details of the collected data was published before^39^.

Variables were coded as follows: maternal education was coded as low (no diploma or primary school), middle (high school), and high (college or university degree); ethnicity was as either European (at least two grandparents were European) or non-European (at least three grandparents were of non-European origin), and parity was coded as primiparous (first child), secundiparous (second child), and multiparous (third or later child)^39,40^.

In the present study, we optimized the protocol using 20 placentas from the ENVIR*ON*AGE birth cohort. Afterwards, 498 bio-banked placental tissue samples were randomly selected from 799 eligible mother-neonate pairs who were recruited between March 1^st^, 2013, and April 1^st^, 2017.

### Sample collection and determination of the wet content

**Figure 3:**
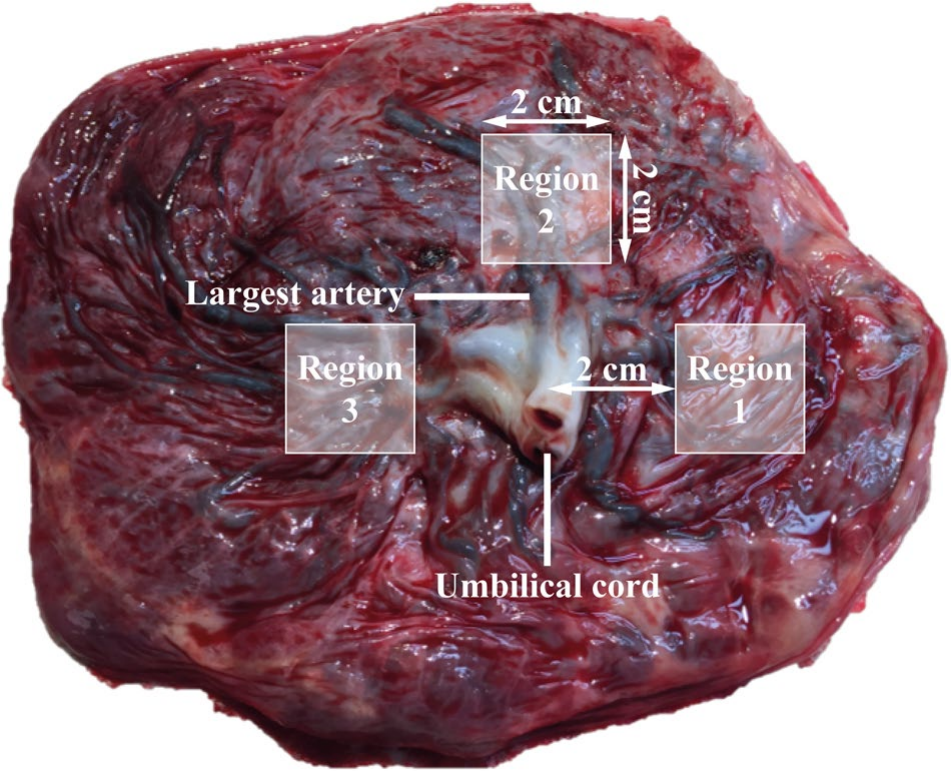
The largest umbilical cord artery on the foetal side of the placenta was used as reference location to identify the placental entry. Three biopsies of 2 by 2 cm were taken at a distance of 2 cm from the umbilical cord and treated further (removal of membranes and excess blood) to obtain three samples of each placenta.

Whole placentas were deep frozen in plastic containers at −20 °C within 10 minutes after delivery. After minimally thawing, placental tissue biopsies were taken at three standardized regions (Fig. 3). To this end, the placenta was placed with the foetal side upwards and orientated with the largest blood vessel originating from the umbilical cord facing away from the researcher. Biopsies of two by two cm were taken at a distance of two cm away from the umbilical cord by cutting with a ceramic knife. For each biopsy, the membranes were cut away and excess blood was removed from the tissue by squeezing and rubbing it against Grade 54 filter paper (GE Healthcare, Chicago, USA). As such, we made sure that the maternal and foetal sides of the tissue were adequately blended to get a homogeneous sample, representative for the entire region. Three different samples were thus obtained for each placenta and stored separately in metal-free centrifuge tubes (Sterile propylene tubes; VWR, Radnor, USA) at −20 °C until extraction.

One day after delivery, maternal urine samples were collected in metal-free tubes (BD Vacutainer Plastic Urinalysis Tubes, Fisher Scientific, Pittsburgh, USA), and frozen at −80 °C until analysis.

In order to determine whether potential differences in water content between placental samples should be taken into account for the interpretation of the results, a subset of ten placentas was randomly selected to verify the presence of differences in water content. This was assessed by weighing from each separate placental region approximately one gram of tissue in a glass petridish and drying it in an oven (T5050, Heraeus, Hanau, Germany) at 105 °C for 24 hours. After drying, the samples were cooled in a silica gel desiccator for 30 minutes and weighed again. Final dry weight percentages were calculated as an average of the three samples per placenta.

### Standards and chemicals

Stock solutions of 1000 mg/L iodide (as potassium iodide) and 1000 mg/L Te (tellurium) were purchased from Sigma-Aldrich (Saint Louis, USA), and TMAH (99.9999%) at 25% w/v from VWR (Radnor, USA). Intermediate solutions of 1 mg/L iodide in 0.5% TMAH and 10 mg/L Te in 0.5% TMAH were prepared through dilution of the stock solutions with double-distilled water. Calibration blanks and final calibration standards of 0.5, 1, 2, and 5 *μ*g/L iodide were prepared after additional dilution of the intermediate solution with double-distilled water and addition of Te to a concentration of 500 *μ*g/L (as an internal standard for ICP-MS measurements). The multi-elemental Elan DRC II smart tuning solution (Perkin Elmer, Massachusetts, USA) was used for daily tuning of the ICP-MS.

### Sample preparation

Placental samples were thawed, approximately 500 mg of tissue was weighed into 50 ml DigiPREP tubes (SCP SCIENCE, Quebec, Canada) to which was added 0.5 ml of 25% TMAH solution and 2.5 ml double-distilled water. Subsequently, the tubes were heated for 3 hours at 90 °C in a DigiPREP block digestion system (SCP SCIENCE, Quebec, Canada). After cooling to room temperature, further sample preparation included:

1. An additional dilution step to a final concentration of 0.5% TMAH solution with addition of Te to a final concentration of 500 *μ*g/L Te;
2. Centrifugation of the diluted samples for 15 minutes at 10,000 × g;
3. And filtering the supernatant through a 0.45 *μ*m filter (Merck & Co., New Jersey, United States).

Sample solutions, which had a final dilution factor of 100, were stored at 4 °C until ICP-MS analysis. All analyses were done within 6 days of sample preparation.

### Iodine analysis with ICP-MS

The ICP-MS used for the determination of total iodine concentrations (I_tot_) in the sample solutions was an Elan DRC II (Perkin Elmer, Massachusetts, USA). Iodine was measured on mass ^127^I, with ^125^Te as an internal standard. Details on the conditions of the ICP-MS system are presented in Table 3.

**Table 3:** Parameters of the ICP-MS measurement system.

Instrument	Parameter
ICP-MS instrument:	ELAN DRC II
Nebulizer gas flow:	0.94 L/min
Cool gas (plasma gas):	15 L/min
Auxiliary gas:	1.8 L/min
Reaction gas:	Ar at 4 bar
Sample introduction:	micromist all-quartz nebulizer

### Determination of method performance characteristics and quality assurance

#### LOD and LOQ

The LOQ of the method is the lowest level of analyte that can be determined with an acceptable performance, while LOD refers to the level of the analyte where its detection becomes problematic (i.e. not distinguishable from zero)^41^. LOD and LOQ were here calculated as three and ten times the standard deviation of ten procedure blank samples, respectively. As no iodine-free placental samples exist, no matrix matched LOQ could be calculated.

#### Trueness and recovery

Trueness is a theoretical concept, expressing how close the mean of infinite number of results produced by the method is to a reference value. It can be assessed in practice by calculating the relative recovery compared to the reference value (in %). This reference value may, for example, be the concentration of the analyte cited on the certificate of a (certified) reference material, or the intentionally added (spiked) analyte concentration that is detected by the method (i.e. spike recovery)^41^. Since no placental tissue with a certified iodine concentration was found on the market, recovery was assessed by spiking three placental samples to three different levels (75 *μ*g/kg, 150 *μ*g/kg, and 300 *μ*g/kg) using a stock solution of 1000 *μ*g/kg iodine. Three independent replicates per placenta were spiked for each concentration level, as well as an unspiked sample per placenta. Mean recovery was expressed as a percentage of the theoretical concentration. In addition, the bovine liver reference material SRM 1577 (I = 234 ± 31 *μ*g/kg) was added to each analytical series. However, since the reference values presented on the certificate were highly variable between laboratories and measurement techniques used (range 180–280 *μ*g/kg), and because these measurements date back to the 1980s, we used this material for precision determination, rather than for trueness.

#### Precision

Precision is a measure of how close results of the method are to one another when the method is applied on several occasions on the same sample^41^. Repeatability (within-day variation) and intermediate precision (within-lab reproducibility) were determined based on results of the reference material SRM 1577 that was analyzed on 12 different days in two independent replicates. These precision data were confirmed for placental samples based on the analysis of a random placental sample in triplicate on 3 different days. Repeatability standard deviation (s_r_), between-day standard deviation (s_d_) and intermediate precision standard deviation (s_ip_) were calculated via one-way analysis of variance and using the equations below. MSW is the mean squares within days, MSB is the mean squares between days, and n is the number of measurements per day (3 replicates). Relative deviations (expressed in %) were obtained by expressing the corresponding standard deviations as a percentage of the mean measured values:

1 $${s}_{r}=\sqrt{MSW}$$

2 $${s}_{d}=\sqrt{\frac{MSB-MSW}{n}}$$

3 $${s}_{ip}=\sqrt{{s}_{r}^{2}+{s}_{d}^{2}}$$

#### Quality control measures

Each analytical batch included internal quality control measures such as two procedure blanks and a reagent blank as a monitor for possible cross-contamination, a rinsing step between all samples to avoid memory effects, and a quality control check every 20 samples to allow verification of potential instrument drift. A series of acceptance criteria were applied to each batch, including calibration blank value below LOQ/2, procedure blank below the LOQ, and drift below 10%. Additionally, the mean measured value of the reference material SRM 1577 in each batch was evaluated against the value for the MS technique on the certificate (280 ± 10 *μ*g/kg; mean ± measurement uncertainty) with an accepted deviation of maximum 15%.

### Determination of intra- versus interplacental differences in iodine concentrations

In order to gain insight in potential differences in iodine concentrations between the three different placental regions, and to find out whether a single sample of one region could be sufficiently representative for the entire placenta, a random selection of twenty placentas was made to determine the inter- and intraplacental variations. For each of the three sampled regions of these twenty placentas, the iodine concentrations were analyzed and the difference in variation within and between the three sampled regions was statistically evaluated (See paragraph *Statistical analysis*).

### Determination of iodine in 498 placentas of the ENVIR*ON*AGE birth cohort

Based on the assessment of intra-placental variations in iodine concentrations, we decided to pool for each placenta the three sampled regions. For each of these regions, approximately 165 mg was taken and pooled to get a final weight of about 500 mg of placental tissue per placenta. Further processing was done as described in the paragraphs *Sample preparation* on page 6 and *Iodine analysis with ICP-MS*.

### Statistical analysis

The difference in the between- and within-variation of the three sample regions was calculated on the subset of twenty placentas using the intra-class correlation coefficient (ICC) from the variance components of mixed models. Compared to simple linear regression models, the mixed models take regional differences into account for each individual placenta and calculate the proportion of variation that is explained by the variance between individuals^42^. The ICC reflects the variability within or between the placentas. With the Friedman test, the significance of this variability was assessed. Maternal age was investigated via generalized linear models. For the different classes of maternal education level (coded as ‘low’ [no diploma or primary school], ‘middle’ [high school], and ‘high’ [college or university degree]), gestational thyroid status (coded as ‘normal’, ‘hypothyroidism’, and ‘hyperthyroidism’), neonate’s sex (coded as ‘male’ or ‘female’), parity (coded as ‘one’, ‘two’, or ‘at least three’ children), and ethnicity (classified based on the native country of the neonates’ grandparents as either ‘European’ [at least two European grandparents] or ‘non-European’ [at least three grandparents were of non-European origin]) we analysed possible differences in the iodine content via means of the Pearson’s one way Analysis of Variance test (ANOVA). We used Duncan’s new multiple range test as a post-hoc test. Database management and statistical analysis were performed with the SAS software, version 9.4 (SAS Institute, Cary, NC, USA). Mean ± standard deviation (SD) is given for continuous variables and the proportion for categorical variables. The significance level was set at p < 0.05.

## Supplementary information

Supplementary information.
