# Supplementary material for: Variability of iodine concentrations in the human placenta
Source: Sci Rep. 2020 Jan 13;10:161. doi: 10.1038/s41598-019-56775-3 (PMC6957482; doi:10.1038/s41598-019-56775-3)
Supplement: Supplementary file 1 — Supplementary information. [file 41598_2019_56775_MOESM1_ESM.pdf]

Supplemental Table S1 – Maternal and neonate characteristics of the current population study (n=498) Data is presented as mean (± SD) for continuous variables, and as number (%) for discrete variables.

**Characteristics**	**Current study (n=498)**
**Maternal**	
Age, years	29.5 ± 4.4
Pre-pregnancy BMI, kg/m²	24.6 ± 4.8
Self-reported tobacco use[1]	
Never smoker	320 (64.4%)
Smoked before pregnancy	130 (26.2%)
Smoked during pregnancy	47 (9.5%
Alcohol consumption	
None	432 (86.8%)
Maximally 1 glass per day	66 (13.2%)
Maternal education[2]	
Low	64 (12.9%)
Middle	163 (32.7%)
High	271 (54.4%)
Gestational thyroid problems	
None	482 (96.8%)
Hypothyroidism	12 (2.4%)
Hyperthyroidism	4 (0.8%)
**Neonate**	
Gestational age, weeks	39.8 ± 1.1
Birth weight, g	3452 ± 436
Birth length, cm	50.3 ± 1.9
Sex	
Male	256 (51.4%)
Female	242 (48.6%)
Ethnicity[3]	
European	437 (87.8%)
Non-European	61 (13.1%)
Parity	
1	261 (52.4%)
2	172 (34.5%)
≥ 3	65 (13.1%)
Season at birth	
Winter (Dec 21st to Mar 20th)	117 (23.5%)
Spring (Mar 21st to Jun 20th)	116 (23.3%)
Summer (Jun 21st to Sep 20th)	139 (27.9%)
Autumn (Sep 21st to Dec 20th)	126 (25.3%)

[1]*data available for 497 mothers*
[2]*Coded as 'low' (no diploma or primary school), 'middle' (high school) and 'high' (college or university degree)*
[3]*Classification of ethnicity is based on the native country of the neonates' grandparents as either European (at least two grandparents were European) or non-European (at least three grandparents were of non-European origin*

Supplemental Table S2 – Maternal and neonate characteristics of the current population study (n=498) and a reference population of births in Flanders, Belgium (born 2002 until 2011; n=606,877). Data is presented as median (10th percentile – 90th percentile) for continuous variables, and as n (%) for discrete variables.

Characteristics	Current study (n=498)	Births in Flanders (n=606,877)[1]
**Maternal**		
Age, years	29.5 (24.0 – 37.0)	29.5 (23.5 – 35.8)
< 25	51 (10.1%)	98,419 (16.2%)
25 – 34	366 (73.6%)	428,781 (70.7%)
35+	81 (16.3%)	79,677 (13.1%)
Maternal education[2]		
Low	64 (12.9%)	58,743 (13.1%)
Middle	163 (32.7%)	183,410 (40.8%)
High	271 (54.4%)	207,563 (46.2%)
**Neonate**		
Birth weight, g	3463 (2845 – 4010)	3360 (2740 – 3965)
Sex		
Male	256 (51.4%)	311,620 (51.4%)
Ethnicity[3]		
European	437 (87.8%)	384,522 (87.7%)
Parity		
1	261 (52.4%)	284,770 (46.9%)
2	172 (34.5%)	210,731 (34.7%)
≥ 3	65 (13.1%)	111,376 (18.4%)

[1]*Cox, B., E. Martens, B. Nemery, J. Vangronsveld and T. S. Nawrot (2013). "Impact of a stepwise introduction of smoke-free legislation on the rate of preterm births: analysis of routinely collected birth data." BMJ 346: f441.*
[2]*Coded as 'low' (no diploma or primary school), 'middle' (high school) and 'high' (college or university degree)*
[3]*Classification of ethnicity is based on the native country of the neonates' grandparents as either European (at least two grandparents were European) or non-European (at least three grandparents were of non-European origin*

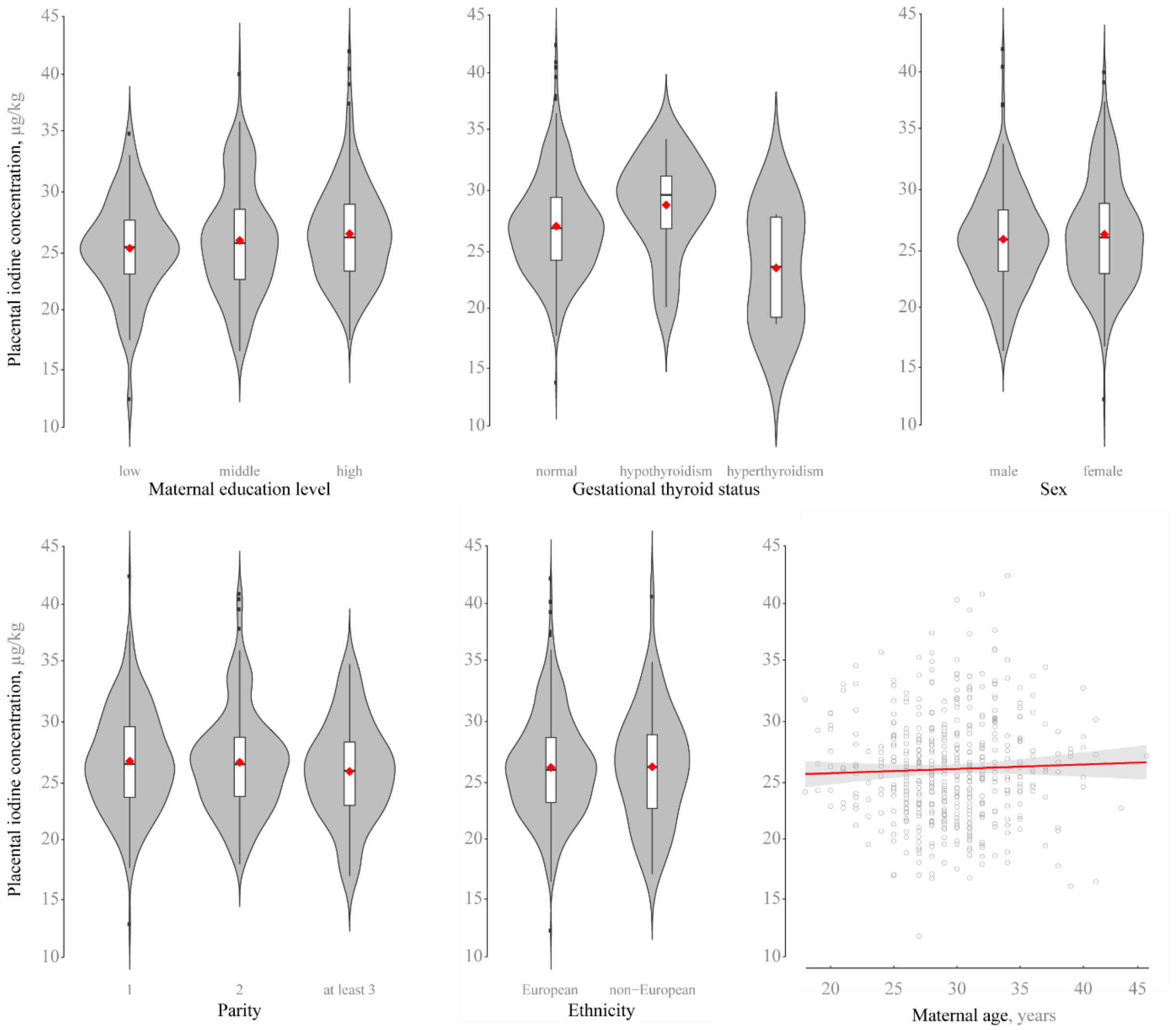

Supplemental Figure S1 – Violin plots of the placental iodine concentration (in µg/kg) for each level of maternal education, gestational thyroid status, neonates' sex, parity, and ethnicity; and a scatter plot of placental iodine concentration (in µg/kg) in association with maternal age (in years). Maternal education level was coded as 'low' (no diploma or primary school), 'middle' (high school), and 'high' (college or university degree), gestational thyroid status was coded as 'normal', 'hypothyroidism', and 'hyperthyroidism', neonates' sex ('male' or 'female'), parity was coded as 'one', 'two', or 'at least three' children, and ethnicity was classified based on the native country of the neonates' grandparents as either 'European' (at least two European grandparents) or 'non-European' (at least three grandparents were of non-European origin). Pearson's one way Analysis of Variance test (ANOVA) was used to test for differences in the iodine content, with the Duncan's new multiple range test as a post-hoc test. Maternal age was investigated via generalized linear models. No significant differences in the placental iodine content were observed for maternal age ($p = 0.42$), maternal education ($p = 0.09$), gestational thyroid status ($p = 0.08$), neonates' sex ($p = 0.28$), parity ($p = 0.34$), or ethnicity ($p = 0.93$).

Within the violin plot, along the boxplot, the grey area represents the density plot (the width is indicative for the frequency). The red diamond in the violin plots indicates the mean per observed group.

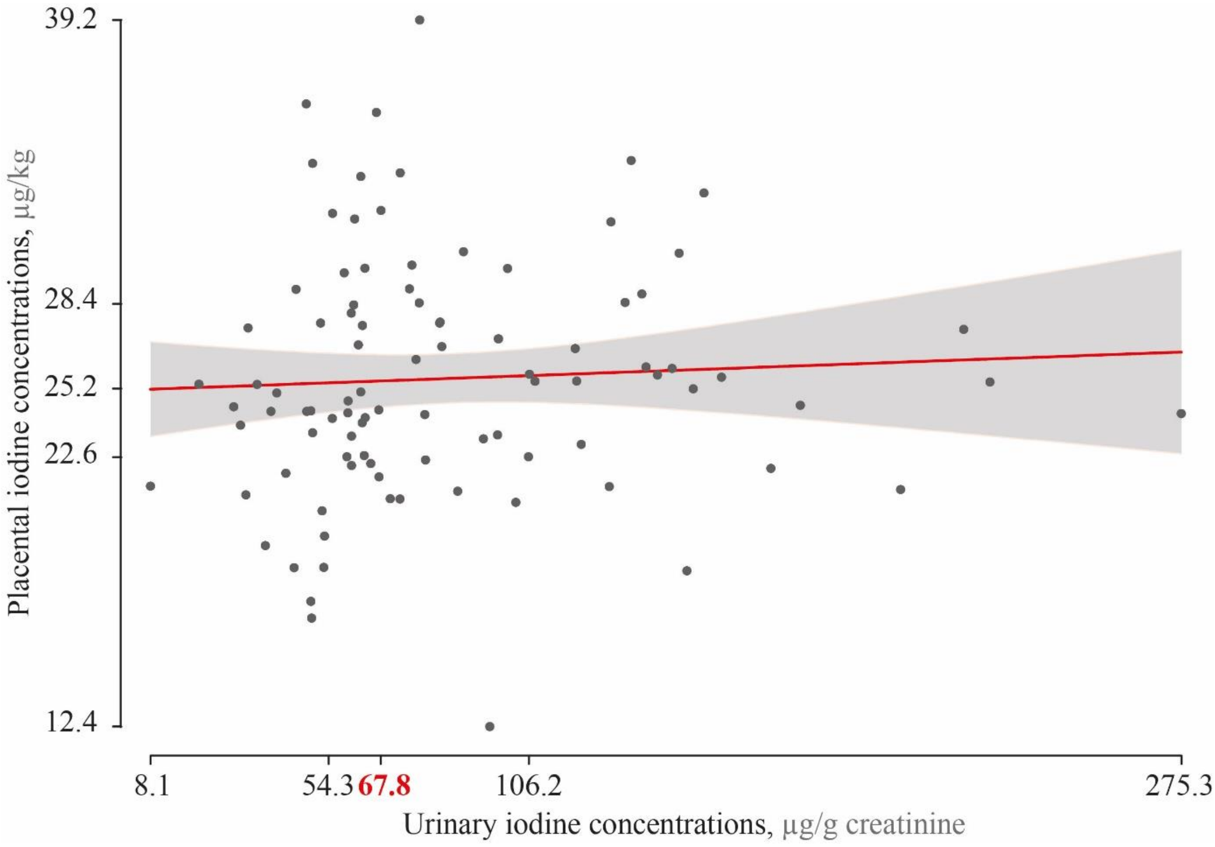

Supplemental Figure S2 – Association between iodine in urine and the placenta. No significant association was observed between the placental and urinary iodine concentrations in a subgroup of 96 participants ($p = 0.98$). The iodine concentrations ranged from 12.4 to 39.2 µg/kg in the placenta, and from 8.1 to 275.3 µg/g creatinine in the urine. Median values were 25.2 µg/kg and 67.8 µg/g creatinine (or 55.7 µg/L when not corrected for creatinine) in the placenta and urine, respectively. Iodine concentrations in urine were determined by ICP-MS (ELAN DRCII, Perkin Elmer) after 1/100 dilution in 0.5% TMAH and using ^{125}Te as an internal standard. The certified reference material NIST 3668 level I was added to the analytical series and showed a mean trueness of 94.1 ± 2.1%.

A concentration of 150 µg/L urinary iodine concentration marks the WHO population threshold for adequate iodine intake. A median value of 55.7 µg/L clearly indicates that the current population, as assessed by a subset of 96 participants, was iodine deficient.

WHO (2013): *Urinary iodine concentrations for determining iodine status deficiency in populations. Geneva, World Health Organization. URL: https://www.who.int/vmnis/indicators/urinaryiodine/en/*
